# Amphiregulin Regulates Melanocytic Senescence

**DOI:** 10.3390/cells10020326

**Published:** 2021-02-05

**Authors:** Michaela Pommer, Silke Kuphal, Anja K. Bosserhoff

**Affiliations:** 1 Institute of Biochemistry, Emil Fischer Center, University of Erlangen-Nürnberg, 91052 Erlangen, Germany; Michaela.pommer@fau.de (M.P.); silke.kuphal@fau.de (S.K.); 2 Comprehensive Cancer Center (CCC) Erlangen-EMN, 91052 Erlangen, Germany

**Keywords:** AREG, amphiregulin, normal human primary melanocytes, oncogene induced senescence, OIS

## Abstract

Oncogene-induced senescence (OIS) is a decisive process to suppress tumor development, but the molecular details of OIS are still under investigation. Using an established OIS model of primary melanocytes transduced with BRAF V600E and compared to control cells, amphiregulin (AREG) was shown to be induced. In addition, AREG expression was observed in nevi, which by definition, are senescent cell clusters, compared to melanocytes. Interestingly, treatment of melanocytes with recombinant AREG did induce senescence. This led to the assumption that extracellular AREG has an important function in this process. Inhibition of the epidermal growth factor receptor (EGFR) using Gefitinib identified AREG as one of EGFR ligands responsible for senescence. Furthermore, depletion of AREG expression in senescent BRAF V600E melanocytes resulted in a significant reduction of senescent melanocytes. This study reveals AREG as an essential molecular component of signaling pathways leading to senescence in melanocytes.

## 1. Introduction

Usually, strongly activated oncogenes induce the transition from normal cells to aggressively growing tumor cells that proliferate in an unrestrained manner. However, in many cancers, this activation may act as genetic stress and causes an irreversible growth arrest in the affected cells, which is named oncogene-induced senescence (OIS) [1]. OIS is induced by the activation of oncogenes coding for BRAF, AKT, E2F1, and cyclin E [2]. Senescent cells show an increased activity of senescence-associated beta-galactosidase (SA β-gal) [3,4], a fried-egg cell morphology, as well as strong changes in chromatin organization [4] and the activation of specific signaling pathways [5]. OIS is generally hypothesized to be important against early oncogenic events by restricting the proliferation of cells with oncogenic mutations [6].

Interestingly, senescent cells remain metabolically active and secrete cytokines (interleukin (IL)-6 and IL-8), chemokines, growth factors (e.g., transforming growth factor-β (TGF-β)), and proteases [7]. These secretory molecules characterize the so-called senescence-associated secretory phenotype (SASP). The DNA damage response (DDR) in OIS triggers histone H2AX phosphorylation and thereby the induction of the SASP phenotype. The SASP supports tumor suppression, leading to the stabilization of the senescent phenotype and restraining tumor growth [8]. However, studies found that the SASP can reduce the efficacy of cancer therapy [9], which makes it a double-edged sword.

In humans, OIS is well documented in the development of melanocytic nevi. Mutation of BRAF (like BRAF^V600E^) in melanocytic cells leads first to cell proliferation, followed by growth arrest, induction of tumor suppressors including p16INK4A and p21CIP1 (p21SDI1, p21WAF1, gene *CDKN1*), and SA β-gal expression, as senescence markers in nevi [10,11]. In experimental cell culture settings, senescence is forced by viral transduction of an expression construct of the BRAF^V600E^ mutant protein in human melanocytes [12,13,14]. In a previous study, we used this experimental model and found melanoma inhibitory activity (MIA) as important factor maintaining senescence in melanocytes [Feuerer12]. Originally, MIA is strongly expressed and secreted by melanoma cells [15,16,17]. However, studies have found an additional role of MIA in OIS.

In this manuscript, we present the secreted molecule amphiregulin (AREG, Schwannoma-derived growth factor (SDGF)), which is upregulated in the above-mentioned senescence model. AREG is part of the epidermal growth factor (EGF) growth factor family, like heparin-binding EGF-like growth factor (HB-EGF), TGF-α, epiregulin (EPR), and the neuregulins 1–4, all binding to the epidermal growth factor receptor (EGFR, or ErbB1). AREG is described to have multiple functions. It was shown to inhibit the proliferation of some cancer cells, while inducing the proliferation of normal cells such as fibroblasts [18]. The amphipathic role of AREG is difficult to analyze because of the diverse intracellular and extracellular localization of the protein. On one side, AREG was found extracellularly after ectodomain shedding (resulting in molecules of different sizes) and on the other side it can be found in the membrane, nucleus, and cytoplasm [19].

In previous studies, it was shown that AREG is released from the cell membrane of senescent cells by the activated metalloprotease A disintegrin and metalloproteinase 17 (ADAM17) via shedding [7]. This manuscript aims at characterizing whether AREG and, especially, its secreted form have an important role in skin melanocytes.

## 2. Materials and Methods

### 2.1. Cell Lines and Cell Culture Conditions

As described previously [20], normal human epidermal melanocytes (NHEM, Lonza, Basel, Switzerland) were grown in MGM-4 BulletKit medium (Lonza). NHEM from different donors, at passage 7 or 8 were used. HEK293T cells were a generous gift of Prof. Stephan Hahn (Ruhr-Universität Bochum, Germany) and were cultivated in high-glucose Dulbecco’s modified Eagle’s medium (DMEM) supplemented with penicillin (400 U/mL), streptomycin (50 μg/mL), and 10% fetal calf serum (all from Sigma-Aldrich, München, Germany). Both cell lines were incubated at 37 °C in a 5% CO_2_ humified atmosphere. Cells were treated with Gefitinib (10 µM) (Absource Diagnostics, Munich, Germany (ZD1839)) twice in 72 h. Cells were treated with recombinant AREG (100 ng/mL) (Sigma Aldrich (SRP6201)) for 24 or 72 h.

### 2.2. Immunofluorescence Staining

As described previously [21], the cells were cultivated in 8-well chamber slides (Corning Incorporated, Corning, USA) and incubated at 37 °C. After 15 min of fixation with 4% paraformaldehyde, the cells were washed three times with PBS and blocked for 1 h with 1% BSA and 0.1% Triton-X-100 in PBS. Subsequently, the cells were incubated with anti-AREG (1:100, sc-74501, Santa Cruz Biotech, Heidelberg, Germany) or anti-PML antibody (1:200, sc-966, Santa Cruz Biotech, Heidelberg, Germany) for 1 h at room temperature, and after three washing steps, the secondary Cy3 antibody (1:500, Biozol, Eching, Germany) was applied for 1 h. Finally, the cells were incubated in DAPI solution (Merck KGaA, Darmstadt, Germany) in 1% BSA/PBS for 30 min, followed by washing three times with PBS. Aqua-Poly/Mount (US Headquarters Polysciences, Warrington, PA, USA) was used as mounting medium. Immunofluorescence staining was analyzed with an IX83 microscope (Olympus, Hamburg, Germany).

### 2.3. Senescence-Associated β-Galactosidase Staining

For senescence-associated β-galactosidase staining [3], a staining kit from Cell Signaling (#9860S) (Cell Signaling Technology, Frankfurt, Germany) was used according to the manufacturer’s protocol [20]. The cells used in this manuscript were seeded in six-well culture plates and were incubated in the staining solution for 10 h. Five microscope images were taken from each treatment, at 20× magnification. All visible cells and SA-β-Gal-positive cells were counted, and the ratio of SA-β-Gal-positive cells to total cells was reported.

### 2.4. Lentiviral Transduction

Lentiviral transduction was carried out as described previously [22]. In short, packaging cells (HEK293T) were transfected with a third-generation vector system. For transfections, pCMVΔR8.2, pHIT G, and the plasmid DNA of interest (copGFP and B-RafV600E) were mixed with DMEM (without phenol red), and subsequently 24 µL Lipofectamine Plus (Thermo Fisher Scientific, Waltham, MA, USA) was added to a final volume of 160 µL (A). Twenty microliters of Lipofectamine LTX (Thermo as) was mixed with 140 µL DMEM (without phenol red) (B). After incubation for 10 min, mixtures A and B were combined, incubated for 30 min at RT, and finally added to HEK293T cells, which had been counted and put in culture the day before in 10 mL of high-glucose DMEM into a 10 cm dish. After incubation for 16 h (37 °C and 5% CO_2_), the cell medium was changed to MGM-4 BulletKit medium (Lonza, Basel, Switzerland). Twenty-four hours later, the lentiviral supernatants were collected and filtered for the subsequent infection of target cells (NHEM at passage 7 or 8). RNA and protein samples were obtained, and all other experiments were performed 7 days after transduction.

### 2.5. Western Blot Analysis

As described previously [20], cell pellets were lysed in 100 µl RIPA buffer (Roche, Mannheim, Germany) for 15 min at 4 °C. Cell fragments were removed by centrifugation (13,000× *g* rpm, 10 min, 4 °C), and the supernatants were collected. Then, 40 µg of total RIPA lysates were loaded on polyacrylamide gels. After separation, the gel was blotted onto a PVDF membrane. Each blot was blocked for 1 h with 5% milk powder/TBS-T and incubated overnight at 4 °C with anti-AREG (1:500, Santa Cruz Biotech, Heidelberg, Germany), anti-PML (1:200, Santa Cruz Biotech, Heidelberg, Germany), anti-H3K9 (1:500, Merck KGaA, Darmstadt, Germany), anti-gamma-H2AX (1:1000, Cell Signaling Technology, Frankfurt, Germany), anti-p21 (1:1000, Abcam, Berlin, Germany), or anti-β-actin (1:5000, Sigma-Aldrich) in 3% MP/TBST. After washing three times with TBST, the membrane was incubated with a horseradish peroxidase-coupled secondary antibody (1:2000, anti-rabbit HRP or anti-mouse HRP, Cell Signaling Technology) for 1 h. The immunoreactions were visualized by ECL staining (Bio-Rad, Feldkirchen, Germany).

For competitive Western blot, 400 ng of AREG antibody was pre-incubated with 4 µg of recombinant AREG (Sigma Aldrich, Germany) for 3 h at 4 °C before overnight incubation with the PVDF membrane.

### 2.6. Enzyme-Linked Immunosorbent Assay (ELISA)

The AREG ELISA was performed as described in the manufacturer’s instructions (Abcam, Berlin, Germany ab222504).

### 2.7. RNA Isolation and Reverse Transcription

Isolation of total cellular RNA from cultured cells and generation of cDNAs by reverse transcription (RT) were performed as described previously [20]. Human skin biopsies of nevi were collected at the Department of Dermatology and the Institute of Pathology, University Hospital Regensburg, Germany. As prescribed by the medical ethical committee of the University Regensburg, Germany, data privacy was protected, and safeguards were put in place to protect the identity of the subjects participating in the study. The sampling and handling of patient material was carried out in accordance with the ethical principles of the Declaration of Helsinki and following IRB approval (protocol code: #12-5152-BO).

### 2.8. Analysis of mRNA Expression

Quantitative real-time PCR (qRT-PCR) analysis of gene expression was performed on a LightCycler 480 system with specific sets of primers, as described previously [23]. Primer sequences were: hβ-Act_735for CTACGTCGCCCTGGACTTCGAGC, hβ-Act_1119rev TGGAGCCGCCGATCCACACGG; hAREG_301forCTCCCGAGGACGGTTCACTA, AREG_483revTCGGCTCAGGCCATTATGCT.

### 2.9. siRNA Transfection

NHEM were transfected with a siPool against AREG (functionally confirmed by siTOOLs Biotech, Planegg/Martinsried, Germany [24]) by using lipofectamine RNAiMAX (Life Technologies, Darmstadt, Germany), as described previously [Dietrich 23]. Si-RNA-Pools consist of multiple siRNAs resulting in efficient target-gene knockdown with minimal off-target effects [24]. After transduction of NHEM with the lentiviral constructs, fresh medium containing the siPools (50 pmol of AREG siRNA (siAREG) or negative control siRNA) was added to the cells. The cells were incubated for 6 h.

### 2.10. Statistical Analysis

Statistical analysis was performed using GraphPad Prism software (GraphPad Software, Inc., San Diego, CA, USA). This software was also used to create the graphs. The results are expressed as the mean ± SEM (range) or percent. Comparison between groups was determined using Student’s t-test. A *p* value < 0.05 was considered statistically significant (n.s.: not significant; *: *p* < 0.05).

## 3. Results

### 3.1. Induction of AREG in Oncogene-Induced Senescence

**Figure 1:**
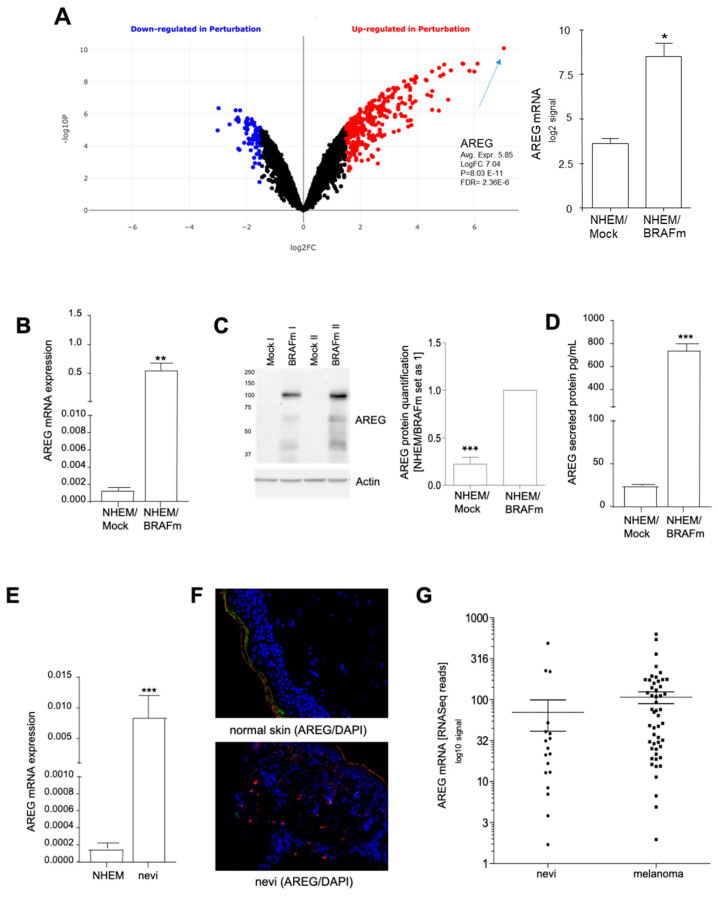
Analysis of amphiregulin (AREG) expression in senescent normal human epidermal melanocytes (NHEM)/BRAFm cells. (**A**–**D**) NHEMs were transduced with lentiviruses expressing oncogenic B-Raf^V600E^ (BRAFm) or copepod green fluorescent protein (copGFP, NHEM/Mock) cDNA. Arrays with *n* = 3 sample pairs were analyzed [12], and the volcano blot was generated via bioinformatical analyses (https://amp.pharm.mssm.edu/biojupies/analyze) (**A**), AREG detection by qRT-PCR (*n* = 11) (**B**), western blot analysis (*n* = 10) (**C**), and ELISA (*n* = 7) (**D**). The expression level of AREG was determined by Western blot analysis 7 days after infection using β-actin as a loading control. The ELISA was performed with cell culture supernatant. (**E**–**G**) AREG expression in vivo was detected in normal melanocytes (*n* = 6) compared to nevi (*n* = 6), using qRT-PCR (**E**); example of immunofluorescence staining of nevi tissue (*n* = 10) and normal skin (*n* = 5) (AREG: red; nucleus: blue) (**F**). AREG expression analyzed by in silico analysis of microdissected nevi (*n* = 23) and primary melanoma tissues (*n* = 57) generated by RNASeq analysis (Accession: GSE112509) (**G**). (*: *p* < 0.05; **: *p* < 0.01; ***: *p* < 0.005).

OIS was experimentally induced in melanocytes (NHEM) by lentiviral transduction of mutant B-RafV600E, and the resulting cells were compared to GFP-transduced cells. This established model system was characterized for the induction of a senescence phenotype and growth arrest by Tran et al. and was further analyzed by our group [14,20]. Subsequent cDNA array analysis using Biojupies (https://amp.pharm.mssm.edu/biojupies/analyze; access date: December 2020, [25]) revealed significantly increased AREG mRNA expression in NHEM expressing B-RAFV600E (NHEM/B-RAFm) compared to control cells (NHEM/Mock) (Figure 1A, *p* = 0.011). The data were confirmed by quantitative RT-PCR (Figure 1B) and Western blot analysis. Western blots of two different transduction experiments are shown as examples in Figure 1C. The densitometry of these Western blots was performed for the ~55 kDa AREG band. Although the competitive Western blot confirmed that different AREG variants are present in melanocytes (~100 kDa, ~55 kDa, and ~40 kDa), in this study we focused on the commonly known variant of 55 kDa (Figure S1A). The 100 kDa variant is very interesting; however, this variant was not shown in the literature before and it is unknown whether it is a splicing variant as suggested by ENSEMBL or corresponds to strongly modified AREG. The secretion of AREG after induction of OIS was analyzed by ELISA. We observed strong induction of AREG protein in the cellular supernatant (Figure 1D).

Prior to focusing on the analysis of AREG function, the localization of the protein was determined. Guerad et al. and other groups reported that AREG can be found in the nucleus [19,26,27,28,29]. This was confirmed by our analysis mainly for the 55 kDa variant. The larger ~100 kDa band was also detected in the cytoplasm (Figure S1B).

AREG expression was confirmed at the mRNA level in vivo, by comparing samples from NHEM and from nevi (*n* = 6). A strong induction of AREG was observed in nevi samples (Figure 1E). AREG expression was confirmed by immunofluorescence staining of nevi tissue versus normal skin, which is negative for AREG staining (Figure 1F). In agreement with the staining pattern of, e.g., p16 [14], not all nevus cells appeared positive. Furthermore, we used an in silico analysis of microdissected nevi tissue (*n* = 23) which was evaluated by RNASeq analysis [30] and confirmed that AREG was expressed in 18 of 23 nevi. This expression level is not significantly different from the RNA expression level in microdissected primary melanomas (Figure 1G).

### 3.2. Role of AREG in OIS Induction

**Figure 2:**
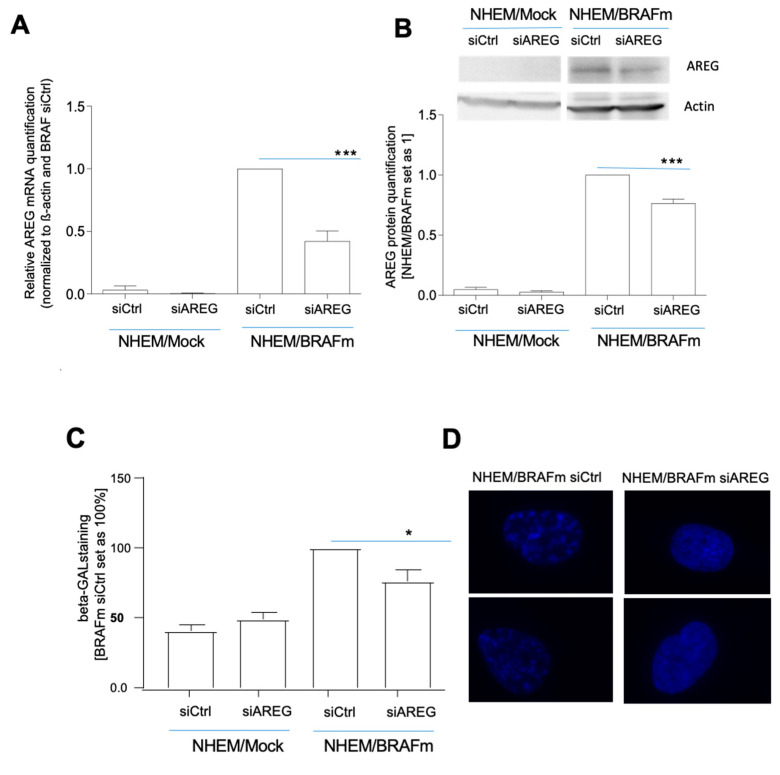

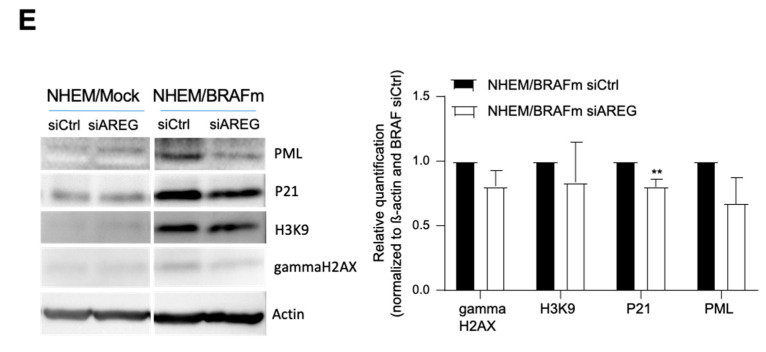
Influence of AREG knockdown on senescence characteristics in NHEM B-Raf^V600E^ cells. NHEM/BRAFm and NHEM/Mock cells were transfected with scrambled siRNA (siCtrl) or siRNA against AREG (siAREG) (*n* = 11). Successful knockdown was validated with qRT-PCR (**A**) and western blot analysis (*n* = 7) (**B**). Light microscopic images of SA-β-Gal staining in NHEM/BRAFm cells with scrambled siCtrl and siAREG were analysed (*n* = 5). The percentages of SA-β-galactosidase-positive cells is shown (NHEM/BRAFm/siCtrl = 100%) (**C**). Images of DAPI staining of two magnified nuclei of NHEM/BRAFm siCtrl cells versus NHEM/BRAFm siAREG-transfected cells. The speckled staining hints to the formation of heterochromatin foci (*n* = 4) (**D**). Senescence indicators were analyzed by Western blot analysis comparing NHEM/Mock cells with NHEM/BRAFm cells transfected with siCtrl and siAREG. The densitometry of the Western blots is shown (right). Only p21 was significantly downregulated at the protein level (*n* = 9) (**E**). (*: *p* < 0.05; **: *p* < 0.01; ***: *p* < 0.005).

To prove the importance of AREG for the induction of senescence, siAREG was used, and downregulation of AREG expression was confirmed at the RNA (Figure 2A) and protein levels (Figure 2B). Although the very strong induction of AREG in senescence was only partially suppressible, we observed a significant reduction in the number of senescent cells after BRAF transduction in siAREG-treated cells compared to siCtrl cells using SA-β-gal staining (Figure 2C). Senescent cells frequently present changes in chromatin structure and form the so-called senescence-associated heterochromatin foci (SAHF). These SAHF result in stable downregulation of pro-proliferative genes and in cell cycle arrest. Changes in chromatin structure were visible in NHEM/BRAFm siCtrl cells, as shown by DAPI fluorescence staining. NHEM/BRAFm siAREG cells revealed reduced changes in the formation of heterochromatin foci compared to siCtrl cells (Figure 2D). The promyelocytic leukemia protein (PML), a protein implicated in cellular senescence in melanoma [14,31], is involved in the development of these SAHF, just like gamma-H2AX. Interestingly, siAREG-transfected NHEM/BRAFm cells showed reduction in PML, p21, as well as gamma-H2AX protein expression (Figure 2E).

### 3.3. OIS induction is Regulated by AREG-Induced Signaling

**Figure 3:**
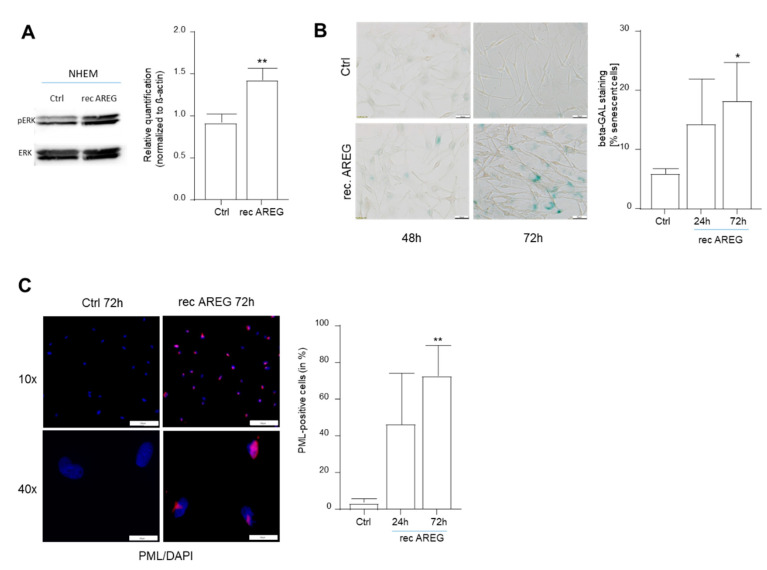
Influence of recombinant AREG (rec AREG) on senescence characteristics in NHEMs. Detection of ERK (loading control) and phospho-ERK by Western blot analysis 30 min after treatment of NHEMs with rec AREG (*n* = 4) (**A**). Light microscopic images of SA-β-Gal staining in NHEMs treated with rec AREG for 24 or 72 h (*n* = 4). The percentages of SA-β-galactosidase-positive cells (blue) were calculated (right). Scale bar: 50 µM (**B**). Representative images of PML immunofluorescence staining of NHEMs treated with rec AREG for 72 h. The panels show overlays of PML (red) and DAPI (blue) staining. The graph shows nuclear accumulation of PML after 24 and 72 h of treatment with rec AREG (*n* = 2). Scale bars upper panel 200 µm. Scale bars lower panel: 100 µm (**C**). (*: *p* < 0.05; **: *p* < 0.01).

To determine whether extracellular AREG regulates senescence via binding to the EGFR receptor, we treated melanocytes with recombinant AREG and analyzed the induction of senescence. To control the treatment, we demonstrated increased phosphorylation of ERK downstream in the EGFR signaling cascade 30 min after AREG treatment (Figure 3A). Treatment of the cells resulted in an increase in SA-β-Gal-positive cells, which was shown to be significant (Figure 3B). Induction of senescence was further supported by the detection of increased levels of nuclear PML (Figure 3C). The experiments with recombinant AREG indicated that soluble/secreted AREG may have a stronger regulatory function in melanocytic senescence than intracellular AREG.

### 3.4. OIS Induction is Modulated by Extracellular AREG

**Figure 4:**
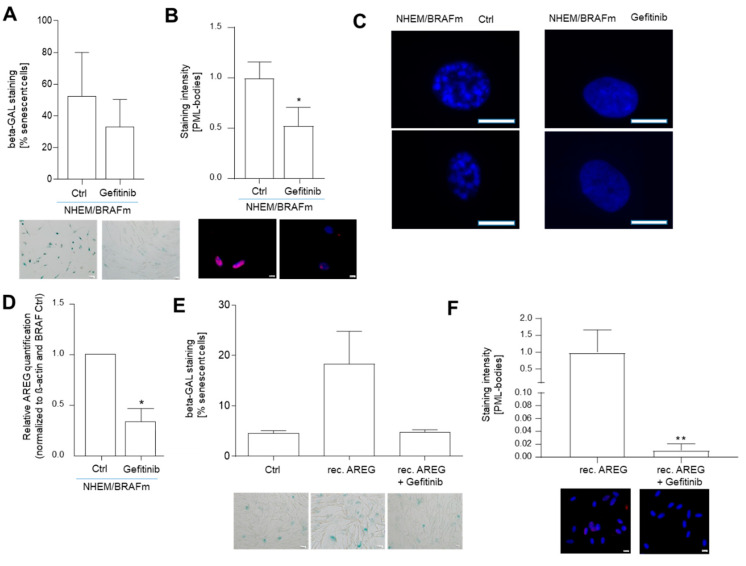
Inhibition of epidermal growth factor receptor (EGFR) with Gefitinib and its influence on extracellular AREG signaling. Light microscopic images of SA-β-Gal staining in NHEM/BRAFm treated with Gefitinib. The percentages of SA-β-galactosidase-positive cells (blue) were calculated (*n* = 3). Scale bar: 50 µM (**A**). Images of PML immunofluorescence staining of NHEM/BRAFm treated with Gefitinib. The panels show overlays of PML (red) and DAPI (blue) staining. The graph shows nuclear accumulation of PML (*n* = 5). Scale bar: 50 µM (**B**). Images of DAPI staining of two magnified nuclei of NHEM/BRAFm Ctrl cells versus NHEM/BRAFm Gefetinib-treated cells. The speckled staining hints to the formation of heterochromatin foci (*n* = 5). Scale bar: 25 µM (**C**). AREG expression after Gefitinib treatment was analyzed in NHEM/BRAFm cells by Western blot. Shown is the densitometry of the Western blots (*n* = 3) (**D**). Representative light microscopy images of SA-β-galactosidase staining in NHEMs treated with rec AREG alone or in combination with Gefitinib. The percentages of SA-β-galactosidase-positive cells (blue) were calculated (*n* = 4). Scale bar: 50 µM (**E**). Representative images of PML immunofluorescence staining of NHEMs treated with rec AREG alone or in combination with Gefitinib. The panels show overlays of PML (red) and DAPI (blue) staining. The graph shows the quantification of the nuclear accumulation of PML (*n* = 3). Scale bar: 50 µm (**F**). (*: *p* < 0.05; **: *p* < 0.01).

Next, we inhibited EGF receptor signaling in NHEM/BRAFm cells using Gefitinib. The inhibition of this signaling cascade resulted in reduced SA-β-gal-positive cells compared to control (Ctrl) BRAFm NHEMs (Figure 4A). Again, the reduction of senescence was supported by the reduced intensity of nuclear PML staining (Figure 4B). Furthermore, DAPI staining of the nuclei hinted to the formation of heterochromatin foci only in NHEM/BRAFm cells with respect to NHEM/BRAFm treated with Gefitinib (Figure 4C). Interestingly, the treatment also resulted in the inhibition of AREG expression, as shown by Western blot and subsequent densitometric analysis (Figure 4D), suggesting an autoregulatory loop.

To control that the effect of Gefitinib was dependent on AREG, as Gefitinib is a general EGFR inhibitor, NHEM were incubated with recombinant AREG (rec AREG) to induce senescence (as shown in Figure 3) or treated simultaneously with rec AREG and the inhibitor Gefitinib. This combined treatment led to a reduction of AREG-induced senescence measured by SA-β-galactosidase to the initial value of the untreated control (Figure 4E). Induction of senescence with rec AREG and reduction of senescence induction by treatment with AREG and Gefitinib were further supported by staining for nuclear PML (Figure 4F).

## 4. Discussion

BRAF^V600E^ was identified as a common mutation in human cancer, predominantly melanoma [27]. Interestingly, the same BRAF^V600E^ mutation was also detectable in the majority of nevi [10]. In spite of the presence of the oncogenic BRAF molecule, nevi are normally associated with an extremely low proliferative activity and rarely progress into melanoma. This is explainable by the fact that the melanocytic nevus was the first human lesion in which OIS was defined as a process that prevents malignant progression to melanoma [11,28]. After this observation, various research groups designed cellular models to investigate OIS, and diverse molecules were found to be involved in this process in healthy and cancerous cells.

Goel et al. created a transgenic mouse in which melanocytes specifically express BRAFV600E, so to mimic the situation found in human nevi [32]. Interestingly, BRAFV600E mice developed skin hyperpigmentation, hyperproliferation of melanocytes, and, at a low frequency, melanomas. Regarding the rare melanoma development, the group revealed that OIS serves as an important molecular barrier to malignant transformation [32,33].

In further studies, SASP components (cytokines or chemokines), as well as diverse growth factors, were found to be produced by senescent human cells [34,35]. For example, in stromal cells (comprising mainly fibroblasts), AREG and Tumor necrosis factor receptor 1 (TNFRI) and their role in senescence were investigated. It was revealed that in different models of senescence, ADAM17 is activated and promotes the release of AREG and TNFRI from the cell surface of senescent cells. The levels of the paracrine-acting proteins FGF-7, HGF, and AREG were also further observed to be elevated in the extracellular environment of senescent prostate fibroblasts [7,29,36]. AREG can also be secreted by mast cells in chronic inflammation and plays a role in the enhancement of immunosuppressive competency of regulatory T cells. If AREG is absent in the environment of T cells, local inflammation cannot be supressed. Tumor antigens can also promote tolerance of the immune system, leading to uncontrolled tumor growth. The available data hint to a link between mast cells and T-reg cells in the tumor microenvironment. Researchers speculate that the efficacy of EGFR-targeting agents used in cancer therapy can be influenced by AREG [37].

Our data are in line with these results and show for the first time that also senescent melanocytes (NHEM/BRAFm) strongly express AREG in vitro and in vivo. Additionally, our experiments showed that senescent melanocytes secrete AREG into their extracellular compartment. Experiments with recombinant AREG also hint to the importance of extracellular AREG in regulating melanocytic senescence. We speculate that secreted AREG acts in an autocrine loop on melanocytes themselves. In future work, it will be interesting to analyze the additional impact of secreted AREG on surrounding cells in the microenvironment. Also the role of nuclear AREG in the skin could be an interesting research subject. Here, we showed that NHEM/BRAFm increased specifically the expression of the ~55 kDa variant of nuclear AREG. Other groups found a correlation between nuclear AREG and chemoresistance in cancer cells [37,38]. If this has relevance in senescent melanocytes or in melanoma is still unclear.

In a further study, our working group observed that lentiviral induction of HuR, an RNA-binding protein, in NHEM/BRAF^V600E^ cells enhanced the proliferative capacity of previously senescent melanocytes and led to an inversion of the senescent state [34]. Beside HuR, also AREG, specifically the reduction of AREG by siRNA in senescent NHEM/BRAFV600E cells, led to a reduction of the number of senescent cells.

If AREG has also an influence on melanoma cells will be the topic of a further study. This was shown for two cancer entities. Xu et al. [29] analyzed the role of AREG mainly in the interplay of human stromal cells derived from the breast and prostate with cancer epithelial cells (PC3, DU145, LNCaP, MCF7, MDA-MB-231, MDA-MB-468, T47D, BT-4). The group showed that targeting AREG secreted from the stromal cells in a senescent state diminished cancer resistance and averted programmed cell death 1 ligand (PD-L1)-mediated immunosuppression [29].

## 5. Conclusions

**Figure 5:**
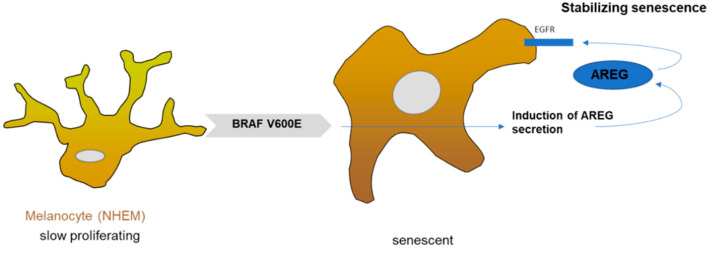
Schematic overview: senescent function of AREG in melanocytes.

The key finding of our study is that AREG expression is induced in senescent melanocytes (Figure 5). Treating proliferating melanocytes with recombinant AREG resulted in senescence, whereas AREG knockdown using siRNA reverted the senescent phenotype of BRAFV600E-transduced melanocytes. In summary, AREG supports and stabilizes OIS in melanocytes, e.g., in nevi, and therefore plays an important tumor-suppressive function in early melanoma development as a component of the SASP.

Melanocytes (NHEM) harboring BRAF^V600E^ mutation showed induced AREG secretion, which led to the binding of AREG to the EGF receptor. A so far unknown signaling cascade regulates AREG-dependent senescence stabilization.

## Data Availability

Data supporting the reported results and links can be found in Feuerer et al. Pigment. Cell Melanoma Res. 2019, 32, 777–791.

## Supplementary Materials

The following are available online at https://www.mdpi.com/2073-4409/10/2/326/s1, Figure S1. Analysis of AREG variants and its AREG localization. AREG antibody was preincubated with recombinant AREG (AB blocking) or not pretreated and used in a western blot of NHEM/BRAFm protein lysate. The ~100 kDa, ~55 kDa, and ~40 kDa AREG bands were weaker using the preincubated AREG antibody (**A**). Fractioning of cell lysates of NHEM/Mock and NHEM/BRAFm cells in nucleus (N), cytoplasma (C), mitochondrium (M) and cytoplasma membrane (CM) and subsequent Western blot analysis of AREG protein. Histone H3 served as a nuclear control, GAPDH and β-actin as cytoplasm controls. Two different variants of AREG (100 kDa, 55 kDa) are shown (**B**).
