# Supplementary material for: Amphiregulin Regulates Melanocytic Senescence
Source: Cells. 2021 Feb 5;10(2):326. doi: 10.3390/cells10020326 (PMC7914549; doi:10.3390/cells10020326)
Supplement: Supplementary file 1 [file cells-10-00326-s001.pdf]

Supplementary Materials

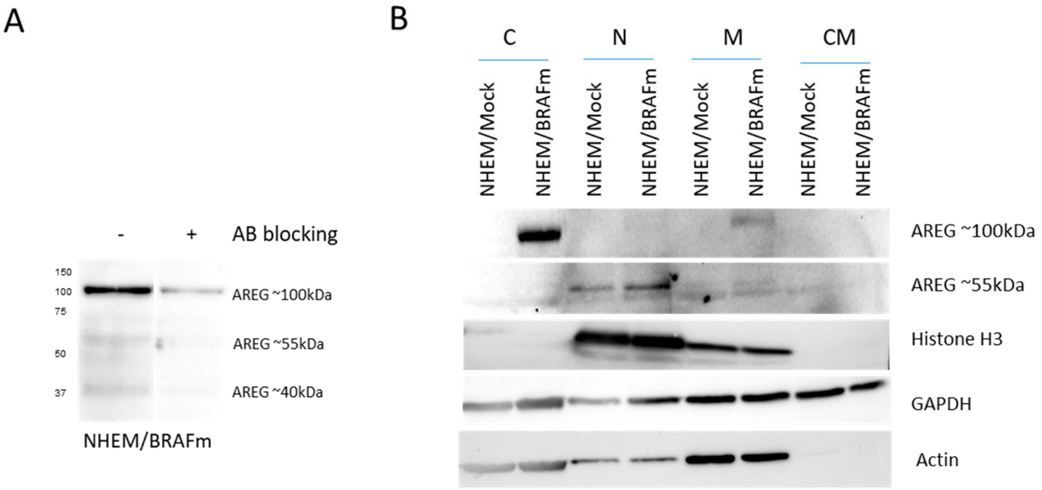

Figure 1. Analysis of AREG variants and its AREG localization. AREG antibody was preincubated with recombinant AREG (AB blocking) or not pretreated and used in a western blot of NHEM/BRAFm protein lysate. The ~100kDa, ~55kDa, and ~40kDa AREG bands were weaker using the preincubated AREG antibody (**A**). Fractioning of cell lysates of NHEM/Mock and NHEM/BRAFm cells in nucleus (N), cytoplasma (C), mitochondrium (M) and cytoplasma membrane (CM) and subsequent Western blot analysis of AREG protein. Histone H3 served as a nuclear control, GAPDH and β-actin as cytoplasm controls. Two different variants of AREG (100kDa, 55kDa) are shown (**B**).
